# A Novel Small Molecule 1,2,3,4,6-penta-*O*-galloyl-α-D-glucopyranose Mimics the Antiplatelet Actions of Insulin

**DOI:** 10.1371/journal.pone.0026238

**Published:** 2011-11-02

**Authors:** Rehana Perveen, Kevin Funk, Jean Thuma, Shelli Wulf Ridge, Yanyan Cao, Jan Willem N. Akkerman, Xiaozhuo Chen, Huzoor Akbar

**Affiliations:** 1 Department of Biomedical Sciences, Heritage College of Osteopathic Medicine, Ohio University, Athens, Ohio, United States of America; 2 Molecular and Cellular Biology Program, Ohio University, Athens, Ohio, United States of America; 3 Thrombosis Treatment and Prevention, Department of Clinical Chemistry and Haematology, University Medical Center Utrecht, Utrecht, The Netherlands; University of Medicine and Dentistry of New Jersey, United States of America

## Abstract

**Background:**

We have shown that 1,2,3,4,6-penta-*O*-galloyl-α-D-glucopyranose (α-PGG), an orally effective hypoglycemic small molecule, binds to insulin receptors and activates insulin-mediated glucose transport. Insulin has been shown to bind to its receptors on platelets and inhibit platelet activation. In this study we tested our hypothesis that if insulin possesses anti-platelet properties then insulin mimetic small molecules should mimic antiplatelet actions of insulin.

**Principal Findings:**

Incubation of human platelets with insulin or α-PGG induced phosphorylation of insulin receptors and IRS-1 and blocked ADP or collagen induced aggregation. Pre-treatment of platelets with α-PGG inhibited thrombin-induced release of P-selectin, secretion of ATP and aggregation. Addition of ADP or thrombin to platelets significantly decreased the basal cyclic AMP levels. Pre-incubation of platelets with α-PGG blocked ADP or thrombin induced decrease in platelet cyclic AMP levels but did not alter the basal or PGE_1_ induced increase in cAMP levels. Addition of α-PGG to platelets blocked agonist induced rise in platelet cytosolic calcium and phosphorylation of Akt. Administration of α-PGG (20 mg kg^−1^) to wild type mice blocked *ex vivo* platelet aggregation induced by ADP or collagen.

**Conclusions:**

These data suggest that α-PGG inhibits platelet activation, at least in part, by inducing phosphorylation of insulin receptors leading to inhibition of agonist induced: (a) decrease in cyclic AMP; (b) rise in cytosolic calcium; and (c) phosphorylation of Akt. These findings taken together with our earlier reports that α-PGG mimics insulin signaling suggest that inhibition of platelet activation by α-PGG mimics antiplatelet actions of insulin.

## Introduction

Patients suffering from diabetes are at a greater risk of thrombotic complications [1], [2], [3], [4], [5], [6] and exhibit a much higher incidence of cardiovascular disease as well as an increased rate of mortality due to ischemic heart disease [7]. Platelets from diabetic patients have been shown to exhibit increased adhesion, secretion and aggregation [8], [9], [10], [11], [12], processes that promote thrombotic complication in diabetics. Increased platelet reactivity in diabetic patients plays a critical role in initiation and progression of thrombosis leading to cardiovascular disease, diabetic nephropathy, retinopathy as well as peripheral artery disease [13], [14].

Reports that abnormal platelet function (secretion, aggregation) occurs not only in platelet-rich plasma but also in washed platelets imply that the mechanism(s) of increased platelet reactivity reside within the platelets [14]. It has been shown that insulin inhibits platelet activation [15]. Moreover, the ability of insulin to inhibit platelet function has been shown to be lacking or diminished in insulin-resistant patients [16], [17], [18]. The direct anti-platelet action of insulin is possibly mediated via regulation of adenylyl cyclase [15]. ADP or thrombin, agonists that induce platelet aggregation, lower basal cyclic AMP levels via stimulation of Giα_2_, a G protein that inhibits adenylyl cyclase [19]. PGI_2_ inhibits platelet activation by stimulating adenylyl cyclase and thereby increasing platelet cyclic AMP levels [20].

Platelets contain insulin receptors [21] and binding of insulin to its receptors induces phosphorylation of its β subunits [22], [23]. This is followed by activation of insulin receptor substrate 1 (IRS-1) and subsequent inactivation of Giα_2_. Inhibition of Giα_2_ prevents agonist-induced lowering of cyclic AMP and the rise in cytosolic calcium, two critical signals for secretion and aggregation of platelets [15]. In other words insulin prevents platelet activation by blocking the agonist-induced lowering of cyclic AMP and the increase in calcium level.

Insulin mimetic, small molecules that possess insulin like activity have the potential to act as therapeutic agents for prevention and management of diabetes [24], [25], [26]. L-783,281, a non-peptidyl fungal metabolite binds to insulin receptors and activates glucose transport [27]. We have shown that α-PGG is an orally effective hypoglycemic small molecule that binds to insulin receptors and activates insulin-mediated glucose transport [28], [29]. Here we report that α-PGG inhibits platelet activation by inducing phosphorylation of insulin receptors leading to inhibition of agonist mediated lowering of cyclic AMP, mobilization of cytosolic calcium and phosphorylation of Akt.

## Materials and Methods

### Materials

α-PGG was custom synthesized at Ohio University [28], [29]. Unless otherwise noted, chemicals and reagents were purchased from Sigma-Aldrich (St. Louis, MO). Collagen was obtained from Chrono-Log Corporation (Havertown, PA).

### Methods

#### Collection of blood and preparation of washed platelet suspensions

All experiments using human blood from healthy volunteers were performed according to the protocol approved by the Institutional Review Board (Protocol #07X067) at Ohio University, Athens, Ohio. Each volunteer was required to sign an informed consent form approved by the Institutional Review Board at Ohio University. Experiments utilizing mice blood were conducted according to the protocol approved by the Institutional Animal Care and Use Committee (Protocol#H08-02) at Ohio University Athens, Ohio. Procedures for drawing human blood, isolation of platelet-rich plasma (PRP) and preparation of washed platelet suspensions are the same as reported earlier [30]. The platelet count was adjusted to 3×10^8^ per ml for aggregation studies.

The *ex vivo* anti-platelet actions of α-PGG were investigated in wild type mice. Blood from mice was drawn by cardiac puncture from anesthetized wild type mice as reported earlier [30], [31], 30 min after oral administration of α-PGG or vehicle, into a syringe containing 100 µl of 3.8% trisodium citrate. The PRP was obtained by centrifugation at 90 g for 10 minutes.

#### Release of P-selectin from α-granules, secretion of ATP from the dense-granules and platelet aggregation

P-selectin release was assessed as described earlier [30]. The PRP was incubated with 1 mM aspirin at 37°C for 30 minutes and then platelets were isolated by centrifugation, washed twice and finally resuspended in HEPES-buffered Tyrode's solution without calcium, pH 7.4 containing 0.2% bovine serum albumin and apyrase (0.1 U/ml). Washed platelets (1–1.5×10^6^) were incubated with 10 µl of FITC-conjugated anti-CD62P (P-selectin) antibody solution for 30 minutes at 37°C without stirring. Expression of P-selectin on platelet surface was quantified by flow cytometry (FACSCalibur, Becton-Dickinson) and the Cellquest software program [30], [32]. Secretion of ATP from the dense granules was assessed by a luminescence method using a luciferin/luciferase kit from Chrono-Log Corporation (Havertown, PA) [30]. The luciferin/luciferase reagent was added to platelets one minute prior to addition of thrombin. Platelet aggregation was monitored as reported earlier [30], [33] using a Lumi-Aggregometer at 37°C and a stirring speed of 900 rpm.

#### Measurement of platelet cyclic AMP levels

Washed human platelets were incubated with α-PGG prior to stimulation with thrombin or ADP and platelet cyclic AMP levels were quantified using the procedure detailed earlier [30], [34]. The reaction was terminated by adding an equal volume of ice-cold 12% trichloroacetic acid [30], [35]. The samples were centrifuged in an Eppendorf micro-centrifuge for 5 minutes. The supernatants were collected and washed three times with 5 ml of water-saturated diethyl ether. The platelet cyclic AMP levels were quantified using cyclic AMP enzyme-immunoassay kits from enzyme-immunoassay kits from Cayman Chemical, Ann Arbor, MI, USA.

#### Assessment of phosphorylation of insulin receptors, IRS-1and Akt

Immuno-precipitation and Western blotting of insulin receptors and IRS-1 was performed as described by Ferreira *et al.* [15]. The effect of α-PGG on agonist-induced phosphorylation of Akt was assessed in samples pre-treated with α-PGG and then stimulated with thrombin. The reactions were terminated by adding 5× sample buffer and proteins were separated by SDS-PAGE. Western blots were probed with anti-Akt and anti-p-Akt antibodies.

#### Measurement of Platelet cytosolic calcium

Platelet cytosolic calcium levels were quantified by using Fura 2/AM-loaded platelets. Washed human platelets were incubated with 3 µM Fura 2/AM at room temperature for 30 min. After incubation, platelets were washed twice and resuspended in a modified Tyrode's solution, pH 7.4. Fluorescence was recorded with a Hitachi F-2000 fluorescence spectrophotometer and calcium levels were quantified as described earlier [33], [36].

#### Statistical analysis

Data are expressed as means ± SD or SEM as described in figure legend. A p value of <0.05 indicates statistically significant difference between the control and α-PGG treated samples.

## Results

### Insulin or α-PGG induced phosphorylation of insulin receptors and IRS-1

**Figure 1:**
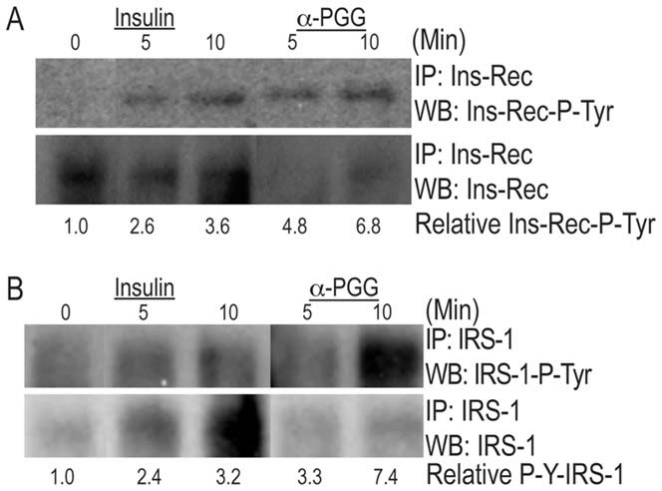
Insulin or α-PGG induced phosphorylation of insulin receptors and IRS-1. Washed human platelets were incubated with insulin (100 nM) or α-PGG (10 µM) for five and ten minutes. The reactions were stopped by adding lysis buffer and the total and phosphorylated insulin receptors (A) and total IRS-1 and phosphorylated IRS-1 (B) were visualized after immuno-precipitation and Western blotting. The phosphorylation of insulin receptors and IRS-1 was quantified by densitometry.

Binding of insulin to its receptors on platelet induces auto-phosphorylation of the β-subunits of insulin receptor and conformational changes that enhance the insulin receptor tyrosine kinase activity leading to phosphorylation of target proteins notably the IRS-1 [15]. We have shown earlier that α-PGG binds to insulin receptors and induces phosphorylation of IR-β in CHO-IR cells [28]. We investigated the possibility that α-PGG may induce phosphorylation of insulin receptors and IRS-1in human platelets. A five minute incubation of washed human platelets with insulin (100 nM) or α-PGG (10 µM) induced 2.6 and 4.8 fold increase in phosphorylation of insulin receptor and 2.4 and 3.3 fold increase in phosphorylation of IRS-1 (Fig. 1). The ability of α-PGG to induce phosphorylation of insulin receptors and IRS-1 suggest that α-PGG mimics the action of insulin on platelets.

### Insulin and α-PGG inhibited in vitro platelet aggregation induced by ADP or collagen

**Figure 2:**
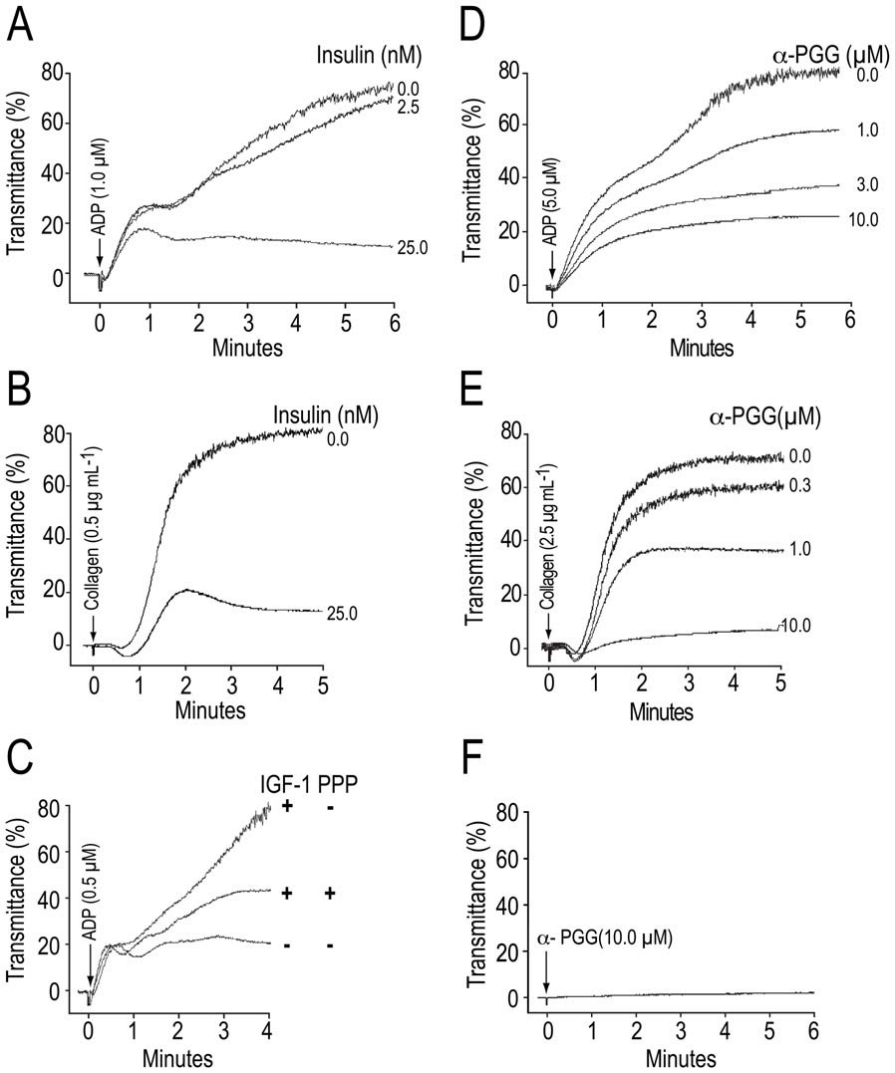
Insulin or α-PGG inhibited *in vitro* platelet aggregation induced by ADP or collagen. Insulin (A, B) or IGF-1 (C) was added to PRP and α-PGG (D, E) was added to washed human platelets five min prior to stimulation with ADP or collagen and aggregation was monitored using an aggregometer from Chrono-Log-Corporation (Havertown, PA, USA). The aggregation tracings are representative of three experiments.

It has been shown that insulin induces phosphorylation of its receptors on platelets and inhibits platelet activation [11], [15], [37]. Based on our data that α-PGG induces phosphorylation of insulin receptors (Fig. 1) we investigated the possibility that α-PGG may mimic the antiplatelet actions of insulin. A five-minute pre-incubation of platelets with insulin (Fig. 2A, B) or α-PGG (Fig. 2D, E) inhibited ADP or collagen induced aggregation. Recently IGF-1 has been shown to enhance platelet aggregation induced by ADP and other agonists [38], [39]. We investigated the effects of IGF-1 and insulin on platelet aggregation in the same platelet preparations and observed that while insulin inhibited ADP (Fig. 2A) or collagen (Fig. 2B) induced platelet aggregation, IGF-1 enhanced platelet aggregation (Fig. 2C). Moreover, the IGF-1 mediated increase in ADP-induced platelet aggregation was reversed by PPP, an IGF-1R antagonist (Fig. 2C). These findings show that insulin inhibits whereas IGF-1 enhances agonist induced platelet activation. Incubation of platelets with α-PGG alone did not induce platelet aggregation (Fig. 2F).

### Evidence that α-PGG inhibited secretion from platelet α- and the dense-granules

**Figure 3:**
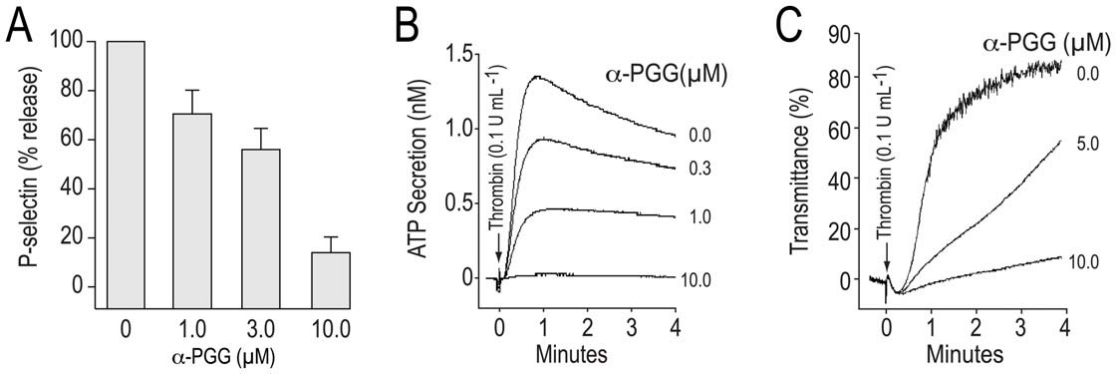
α-PGG inhibited thrombin induced secretion from the α- and dense-granules and platelet aggregation. Washed human platelets were stimulated with thrombin (0.1 U mL^−1^) in the presence or absence of α-PGG and expression of P-selectin (A) and secretion of ATP (B) and platelet aggregation (C) was monitored as detailed in Experimental Procedures. The results are reported as means ± SD for P-selectin expression (n = 3). ATP secretion and aggregation tracings are representative of three experiments.

Secretion from the dense- and α-granules plays critical roles in platelet aggregation. We tested the possibility that α-PGG inhibits platelet aggregation by inhibiting granular secretion. Pre-incubation of human platelets with α-PGG blocked thrombin-induced release of P-selectin from α-granules and secretion of ATP from dense granules as well as aggregation in a concentration-dependent manner (Fig. 3). The ability of α-PGG to inhibit secretion suggests that α-PGG inhibits platelet aggregation, at least in part, by inhibiting secretion.

### α-PGG inhibited agonist induced lowering of cyclic AMP and rise in cytosolic calcium

Agonists such as thrombin or ADP, secreted upon activation of platelets by a variety of agonists, stimulate Giα_2_ [40]. A G protein that inhibits adenylyl cyclase and thereby lowers basal cAMP levels in platelets [15]. Insulin has been shown to inhibit thrombin-induced lowering of cyclic AMP [15]. We investigated the possibility that α-PGG inhibits platelet activation by blocking agonist induced decrease in basal cAMP levels. Incubation of platelets with α-PGG (10 µM) alone did not alter cAMP (pmol/10^8^ platelets) levels (4.64±0.13) as compared to control samples (4.54±0.33). Addition of thrombin (0.1 U ml^−1^) or ADP (10 µM) to platelets decreased cAMP levels by 24% (*p*<0.03) and 22% (*p*<0.02) respectively. A higher concentration of thrombin (0.25 U ml^−1^) has been shown to induce a greater decrease (40%) in cyclic AMP levels [15]. Pre-incubation of platelets with α-PGG completely blocked thrombin- or ADP-induced lowering of cAMP levels (Table 1). Addition of PGE_1_ to washed human platelets increased cAMP level to 24.33±2.86 pmol/10^8^ platelets. Incubation of platelets with α-PGG prior to addition of PGE_1_ did not affect the increase in cAMP (Table 1). These findings suggest that α-PGG affects Gi mediated inhibition but not the Gs mediated activation of adenylyl cyclase.

**Table 1:** α-PGG inhibited ADP- or thrombin-induced lowering of cyclic AMP.

Additions	PBS + PBS	PBS + ADP	PBS α-PGG	α-PGG + ADP	PBS + Thrombin	α-PGG + Thrombin	PBS + PGE_1_	α-PGG + PGE_1_
Mean	4.54	3.44*	4.64	4.28	3.54*	4.58	24.33	25.08
SD	0.33	0.66	0.13	0.30	0.45	0.47	2.86	1.61

ADP (10 µM), thrombin (0.1 U ml^−1^) or PGE_1_ (1 µM) induced changes in platelet cyclic AMP (pmoles/10^8^ platelets) levels were quantified in the presence or absence of α-PGG (10 µM) using enzyme-linked assay kits as described in Experimental Procedures. ADP and thrombin decreased basal cyclic AMP levels by 24% (*p<0.03) and 22% (*p<0.02) respectively. α-PGG blocked ADP and thrombin induced decrease in cyclic AMP levels.

**Figure 4:**
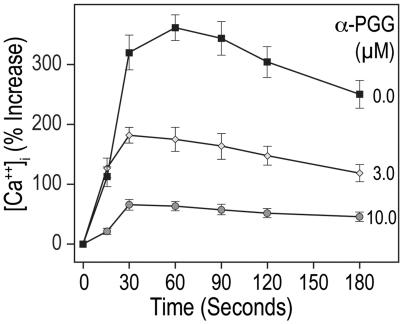
α-PGG inhibited thrombin induced rise in cytosolic calcium. Changes in cytosolic calcium were quantified in Fura2/AM loaded platelets. Platelets were incubated with α-PGG (3 or 10 µM) prior to stimulation with thrombin (0.1 U mL^−1^) and changes in calcium levels were recorded by fluorescence spectrometry as described in Experimental Procedures. The results are reported as means ± SE (n = 4).

Insulin has been shown to inhibit ADP as well as thrombin induced rise in cytosolic calcium in platelets [15]. We investigated the effect of α-PGG on thrombin-induced calcium mobilization using Fura 2/AM loaded washed human platelets. Addition of α-PGG (10 µM) alone to platelets did not alter the basal calcium levels (33.7±4.2 nM). Stimulation of platelets with thrombin (0.1 U ml^−1^) increased the cytosolic calcium level by 362%. Pre-incubation of platelets with α-PGG decreased thrombin induced rise in cytosolic calcium in a concentration-dependent manner (Fig. 4). These data suggest that α-PGG inhibits platelet activation by preventing agonist-induced lowering of cyclic AMP and the rise the cytosolic calcium.

### α-PGG inhibited agonist-induced phosphorylation of Akt

**Figure 5:**
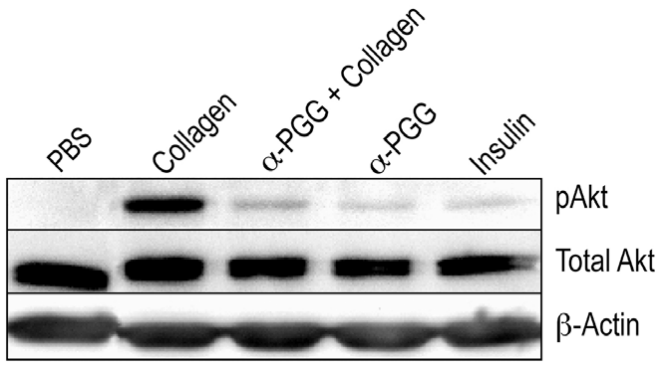
α-PGG inhibited collagen-induced phosphorylation of Akt. Washed human platelets were stimulated with collagen (1.0 µg mL^−1^) in the presence or absence of α-PGG (10 µM). Lysis buffer was added to samples at 6 min to terminate reactions. Total Akt and p-Akt were visualized after PAGE and Western blotting as described in the Experimental Procedures. The β-actin was used as a loading control.

Insulin has been reported to induce phosphorylation of Akt [41]. We therefore investigated the possibility that α-PGG may also induce phosphorylation of Akt. Incubation of washed human platelets with insulin or α-PGG alone for 6 minutes induced phosphorylation of Akt (Fig. 5) without inducing platelet aggregation (Fig. 2F). However, collagen, an inducer of aggregation, induced significantly greater phosphorylation of Akt than insulin or α-PGG (Fig. 5). Agonist induced stimulation of Gi not only lowers cAMP via Giα_2_ but also activates PI3-K via its βγ-subunit, leading to phosphorylation of Akt [35]. We investigated the possibility that α-PGG by blocking activation of Gi also prevents phosphorylation of Akt. Pre-incubation of platelets with α-PGG inhibited phosphorylation of Akt induced by collagen (Fig. 5) or thrombin (data not shown). These findings suggest that α-PGG inhibits platelet activation, at least in part, by inhibiting phosphorylation of Akt.

### Administration of α-PGG inhibited *ex vivo* platelet aggregation induced by ADP or collagen

**Figure 6:**
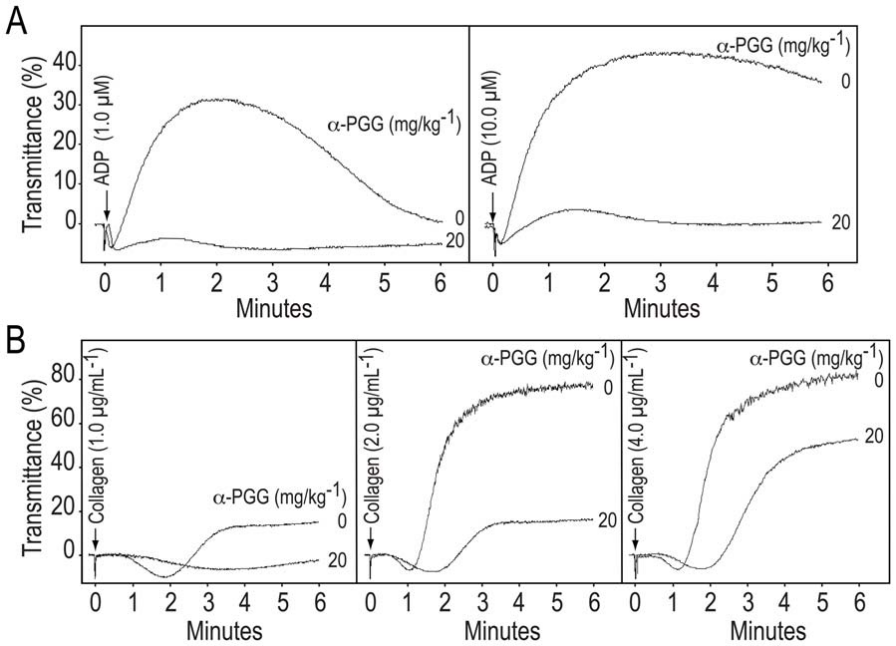
Administration of α-PGG inhibited *ex vivo* platelet aggregation induced by ADP or collagen. A, ADP or B, collagen was added to platelet-rich plasma, prepared from murine blood drawn at 30 min after oral administration of α-PGG (20 mg kg^−1^) or vehicle, to induce aggregation. The aggregation tracings are representative of three experiments.

We have shown earlier that oral administration of α-PGG induces hypoglycemia in db/db mice [28], [29]. We investigated the possibility that oral administration of α-PGG may also inhibit *ex vivo* platelet aggregation. Blood from wild type mice was drawn at 30 minutes after oral administration of α-PGG (20 mg kg^−1^) or saline and platelet aggregation was monitored in platelet-rich plasma. The data in Fig. 6 show that platelet aggregation induced by ADP or collagen is blocked in platelets from mice treated with α-PGG.

## Discussion

This study was undertaken to investigate the effects and the mechanisms of the anti-platelet actions of 1,2,3,4,6-penta-*O*-galloyl-α-D-glucopyranose (α-PGG), an orally effective hypoglycemic small molecule that has been shown to bind to insulin receptors and activate the insulin-induced signaling i.e. phosphorylation of β-subunits of insulin receptor and IRS-1 in CHO-IR cells [28] and RKO cells [42]. The data in this report show that insulin as well as α-PGG induced phosphorylation of insulin receptors and IRS-1 in human platelets (Fig. 1). These findings confirm an earlier report that insulin mediated signaling in platelets involves phosphorylation of insulin receptors and IRS-1[15] and demonstrate for the first time that α-PGG mimics the action of insulin on platelets. The ability of α-PGG to mimic the action of insulin taken together with our earlier findings that α-PGG (a): decreased maximal binding of insulin to its receptors; and (b) displaced insulin from insulin receptors in a concentration-dependent manner with an IC_50_ of 10±1 µM [28] suggest that α-PGG binds to insulin receptors to induce insulin like anti-platelet actions. The possibility that α-PGG binds to insulin receptors is further supported by our findings that both insulin and α-PGG not only induce glucose transport in adipocytes [28] but α-PGG competes with insulin for glucose transport [28]. The ability of HNMPA-(AM)_3_, an inhibitor of the insulin receptor (IR) tyrosine kinase, to block α-PGG-induced glucose transport also implies that α-PGG mediated actions involve binding to insulin receptors [28]. In addition our recent findings that α-PGG induces apoptosis in human colon cancer RKO cells and that the α-PGG induced apoptosis is reduced when RKO cells are treated with siRNA specific to insulin receptor but not the non-specific control siRNA [42] provide conclusive evidence that α-PGG acts by binding to insulin receptors.

Our findings that both insulin and α-PGG inhibited ADP or collagen induced platelet aggregation (Fig. 2) not only are in agreement with reported antiplatelet actions of insulin but also show for the first time that an insulin mimetic small molecule is also capable of inhibiting platelet aggregation. However, α-PGG does not appear to mimic the actions of insulin-like growth factor (IGF-1). Our findings that IGF-1 enhanced ADP-induced platelet aggregation (Fig. 2C) are in agreement with earlier reports that IGF-1 enhances platelets aggregation induced by ADP and other agonists [38], [39]. Moreover, our observation that the IGF-1 mediated increase in ADP-induced platelet aggregation is reversed by PPP, an IGF-1R antagonist, (Fig. 2C) confirms the specificity of the IGF-1 action on platelet activation. These findings suggest that insulin and IGF-1 exert opposite effects on platelet activation by ADP. It takes only 0.2 nM IGF-1, as compared to 160 nM insulin, to displace 50% of the IGF-1 from its receptors [43]. The 800-fold difference between the concentration of IGF-1 and insulin needed to displace IGF-1 from its receptors suggests that insulin and IGF-1 induce signal transduction via their specific receptors. The mechanisms underlying the opposite responses in platelets induced by insulin and IFG-1 remain to be investigated.

Inhibition of secretion from platelet granules diminishes the aggregation response and insulin has been shown to inhibit thrombin-induced release of P-selectin from platelet granules [41]. Our observations that α-PGG inhibited thrombin induced release of P-selection from the α-granules, secretion of ATP from the dense granules as well as aggregation in a concentration-dependent manner (Fig. 3) suggest that α-PGG, at least in part, inhibits platelet aggregation by preventing the release of platelet granular contents.

Agonist-induced platelet activation (secretion, aggregation) involves multiple biochemical pathways leading to a rise in cytosolic calcium and cyclic AMP plays a critical role in regulation of cytosolic calcium levels. Increase in cyclic AMP level inhibits the agonist-induced rise in platelet cytosolic calcium whereas lowering of cyclic AMP facilitates the rise in calcium levels. Insulin has been reported not only to inhibit thrombin-induced lowering of cyclic AMP but also to prevent thrombin-induced rise in platelet cytosolic calcium [15]. Our findings that α-PGG not only induced phosphorylation of IRS-1 but also inhibited thrombin- or ADP-induced lowering of cyclic AMP in platelets (Table 1) and the rise in cytosolic calcium (Fig. 4) further support the possibility that α-PGG mimics the antiplatelet actions of insulin.

Phosphorylation of Akt plays a critical role in the secondary or irreversible platelet aggregation [35], [44], [45], [46]. Insulin and thrombin both induce phosphorylation of Akt in platelets [41]. However, insulin mediated phosphorylation of Akt does not induce platelet aggregation [41]. Our findings that incubation of platelets with α-PGG alone induced phosphorylation of Akt (Fig. 5) but did not induce platelet aggregation (Fig. 2F) suggest that α-PGG induced phosphorylation of Akt in the absence of pro-aggregation signals such as agonist induced lowering of cyclic AMP and or mobilization of calcium is not sufficient to induce platelet activation. The importance of the detectable phosphorylation of Akt, in the absence of any platelet activation (Fig. 2F), induced by α-PGG alone remains to be determined.

In spite of its ability to induce phosphorylation of Akt in the absence of any agonist, α-PGG inhibited collagen-induced phosphorylation of Akt (Fig. 5) as well as platelet aggregation (Fig. 2E). Platelet aggregation agonist such as ADP and thrombin induces activation of Gi leading to its dissociation into Giα_2_ and the βγ sub-units. The Giα_2_ lowers cyclic AMP whereas the βγ sub-units induce phosphorylation of Akt [35]. Our findings that α-PGG inhibits both the lowering of cyclic AMP (Table 1) and phosphorylation of Akt (Fig. 5) suggest that α-PGG inhibits platelet activation by blocking agonist induced activation of Giα_2_.

The *in vitro* anti-platelet activity of α-PGG (Figs. 2,3) taken together with its ability to induce hypoglycemia when given orally [28] suggests that oral administration of α-PGG may be effective in inhibiting platelet aggregation. Our findings that orally administered α-PGG inhibited *ex vivo* platelet aggregation induced by ADP or collagen (Fig. 6) demonstrate that α-PGG is an orally effective anti-platelet agent. The dual hypoglycemic and anti-platelet properties of α-PGG suggest that insulin mimetic small molecules may be developed as orally effective anti-platelet agents for management of thrombotic complications in diabetic patients.

In summary these findings suggest that α-PGG inhibits platelet activation, at least in part, by mimicking the action of insulin i.e. by inducing phosphorylation of insulin receptor and IRS-1 leading to inhibition of agonist-induced lowering of cAMP, rise in cytosolic calcium and the phosphorylation of Akt.

## References

[1] Biondi-Zoccai GG, Abbate A, Liuzzo G, Biasucci LM (2003). Atherothrombosis, inflammation, and diabetes.. J Am Coll Cardiol.

[2] Yngen M, Ostenson CG, Hu H, Li N, Hjemdahl P (2004). Enhanced P-selectin expression and increased soluble CD40 Ligand in patients with Type 1 diabetes mellitus and microangiopathy: evidence for platelet hyperactivity and chronic inflammation.. Diabetologia.

[3] Ferroni P, Basili S, Falco A, Davi G (2004). Platelet activation in type 2 diabetes mellitus.. J Thromb Haemost.

[4] Angiolillo DJ (2006). Tackling the diabetic platelet: is high clopidogrel dosing the answer?. J Thromb Haemost.

[5] Beckman JA, Creager MA, Libby P (2002). Diabetes and atherosclerosis: epidemiology, pathophysiology, and management.. Jama.

[6] Colwell JA, Nesto RW (2003). The platelet in diabetes: focus on prevention of ischemic events.. Diabetes Care.

[7] Orchard TJ, Stevens LK, Forrest KY, Fuller JH (1998). Cardiovascular disease in insulin dependent diabetes mellitus: similar rates but different risk factors in the US compared with Europe.. Int J Epidemiol.

[8] Winocour PD, Perry DW, Kinlough-Rathbone RL (1992). Hypersensitivity to ADP of platelets from diabetic rats associated with enhanced fibrinogen binding.. Eur J Clin Invest.

[9] Turk Z, Flego I, Kerum G (1996). Platelet aggregation in type 1 diabetes without microvascular disease during continuous subcutaneous insulin infusion.. Horm Metab Res.

[10] Angiolillo DJ, Bernardo E, Ramirez C, Costa MA, Sabate M (2006). Insulin therapy is associated with platelet dysfunction in patients with type 2 diabetes mellitus on dual oral antiplatelet treatment.. J Am Coll Cardiol.

[11] Trovati M, Anfossi G, Cavalot F, Massucco P, Mularoni E (1988). Insulin directly reduces platelet sensitivity to aggregating agents. Studies in vitro and in vivo.. Diabetes.

[12] Winocour PD (1992). Platelet abnormalities in diabetes mellitus.. Diabetes.

[13] Vericel E, Januel C, Carreras M, Moulin P, Lagarde M (2004). Diabetic patients without vascular complications display enhanced basal platelet activation and decreased antioxidant status.. Diabetes.

[14] Li Y, Woo V, Bose R (2001). Platelet hyperactivity and abnormal Ca(2+) homeostasis in diabetes mellitus.. Am J Physiol Heart Circ Physiol.

[15] Ferreira IA, Eybrechts KL, Mocking AI, Kroner C, Akkerman JW (2004). IRS-1 mediates inhibition of Ca2+ mobilization by insulin via the inhibitory G-protein Gi.. J Biol Chem.

[16] Westerbacka J, Yki-Jarvinen H, Turpeinen A, Rissanen A, Vehkavaara S (2002). Inhibition of platelet-collagen interaction: an in vivo action of insulin abolished by insulin resistance in obesity.. Arterioscler Thromb Vasc Biol.

[17] Trovati M, Anfossi G (1998). Insulin, insulin resistance and platelet function: similarities with insulin effects on cultured vascular smooth muscle cells.. Diabetologia.

[18] Ferreira IA, Mocking AI, Feijge MA, Gorter G, van Haeften TW (2006). Platelet inhibition by insulin is absent in type 2 diabetes mellitus.. Arterioscler Thromb Vasc Biol.

[19] Dorsam RT, Kunapuli SP (2004). Central role of the P2Y12 receptor in platelet activation.. J Clin Invest.

[20] Cattaneo M, Lecchi A (2007). Inhibition of the platelet P2Y12 receptor for adenosine diphosphate potentiates the antiplatelet effect of prostacyclin.. J Thromb Haemost.

[21] Hajek AS, Joist JH, Baker RK, Jarett L, Daughaday WH (1979). Demonstration and partial characterization of insulin receptors in human platelets.. J Clin Invest.

[22] Lopez-Aparicio P, Rascon A, Manganiello VC, Andersson KE, Belfrage P (1992). Insulin induced phosphorylation and activation of the cGMP-inhibited cAMP phosphodiesterase in human platelets.. Biochem Biophys Res Commun.

[23] Falcon C, Pfliegler G, Deckmyn H, Vermylen J (1988). The platelet insulin receptor: detection, partial characterization, and search for a function.. Biochem Biophys Res Commun.

[24] Moller DE (2001). New drug targets for type 2 diabetes and the metabolic syndrome.. Nature.

[25] Schlein M, Ludvigsen S, Olsen HB, Andersen AS, Danielsen GM (2001). Properties of small molecules affecting insulin receptor function.. Biochemistry.

[26] Manchem VP, Goldfine ID, Kohanski RA, Cristobal CP, Lum RT (2001). A novel small molecule that directly sensitizes the insulin receptor in vitro and in vivo.. Diabetes.

[27] Zhang B, Salituro G, Szalkowski D, Li Z, Zhang Y (1999). Discovery of a small molecule insulin mimetic with antidiabetic activity in mice.. Science.

[28] Li Y, Kim J, Li J, Liu F, Liu X (2005). Natural anti-diabetic compound 1,2,3,4,6-penta-O-galloyl-D-glucopyranose binds to insulin receptor and activates insulin-mediated glucose transport signaling pathway.. Biochem Biophys Res Commun.

[29] Ren Y, Himmeldirk K, Chen X (2006). Synthesis and structure-activity relationship study of antidiabetic penta-O-galloyl-D-glucopyranose and its analogues.. J Med Chem.

[30] Akbar H, Kim J, Funk K, Cancelas JA, Shang X (2007). Genetic and pharmacologic evidence that Rac1 GTPase is involved in regulation of platelet secretion and aggregation.. J Thromb Haemost.

[31] Akbar H, Shang X, Perveen R, Berryman M, Funk K (2011). Gene targeting implicates Cdc42 GTPase in GPVI and non-GPVI mediated platelet filopodia formation, secretion and aggregation.. PLoS One.

[32] Quinton TM, Murugappan S, Kim S, Jin J, Kunapuli SP (2004). Different G protein-coupled signaling pathways are involved in alpha granule release from human platelets.. J Thromb Haemost.

[33] Huzoor A, Wang W, Kornhauser R, Volker C, Stock JB (1993). Protein prenylcysteine analog inhibits agonist-receptor-mediated signal transduction in human platelets.. Proc Natl Acad Sci U S A.

[34] Akbar H, Dean T, Kornhauser R (1993). Increased platelet reactivity to prostaglandin E1 in hypertension is linked with altered signal transduction.. Am J Hypertens.

[35] Li Z, Zhang G, Le Breton GC, Gao X, Malik AB (2003). Two waves of platelet secretion induced by thromboxane A2 receptor and a critical role for phosphoinositide 3-kinases.. J Biol Chem.

[36] Agarwal KC, Clarke E, Rounds S, Parks RE, Huzoor A (1994). Platelet-activating factor (PAF)-induced platelet aggregation. Modulation by plasma adenosine and methylxanthines.. Biochem Pharmacol.

[37] Trovati M, Massucco P, Mattiello L, Mularoni E, Cavalot F (1994). Insulin increases guanosine-3′,5′-cyclic monophosphate in human platelets. A mechanism involved in the insulin anti-aggregating effect.. Diabetes.

[38] Kim S, Garcia A, Jackson SP, Kunapuli SP (2007). Insulin-like growth factor-1 regulates platelet activation through PI3-K{alpha} isoform.. Blood.

[39] Hers I (2007). Insulin-like growth factor-1 potentiates platelet activation via the IRS/PI3Kalpha pathway.. Blood.

[40] Gachet C (2001). ADP receptors of platelets and their inhibition.. Thromb Haemost.

[41] Ferreira IA, Mocking AI, Urbanus RT, Varlack S, Wnuk M (2005). Glucose uptake via glucose transporter 3 by human platelets is regulated by protein kinase B.. J Biol Chem.

[42] Cao Y, Evans SC, Soans E, Malki A, Liu Y (2011). Insulin receptor signaling activated by penta-O-galloyl-alpha-D: -glucopyranose induces p53 and apoptosis in cancer cells.. Apoptosis.

[43] Hartmann K, Baier TG, Loibl R, Schmitt A, Schonberg D (1989). Demonstration of type I insulin-like growth factor receptors on human platelets.. J Recept Res.

[44] Kim S, Jin J, Kunapuli SP (2004). Akt activation in platelets depends on Gi signaling pathways.. J Biol Chem.

[45] Kim S, Mangin P, Dangelmaier C, Lillian R, Jackson SP (2009). Role of phosphoinositide 3-kinase beta in glycoprotein VI-mediated Akt activation in platelets.. J Biol Chem.

[46] Weng Z, Li D, Zhang L, Chen J, Ruan C (2010). PTEN regulates collagen-induced platelet activation.. Blood.

